# The role of host soluble inflammatory mediators induced by the BCG vaccine for the initiation of in vitro monocyte apoptosis in healthy Brazilian volunteers

**DOI:** 10.1186/s12950-015-0105-0

**Published:** 2015-10-29

**Authors:** Jessica Lima, Mariana Siqueira, Thaíze Pedro, Carlos Ponte, Leandro Peres, Suelen Marinho, Luíz R. Castello-Branco, Paulo R. Z. Antas

**Affiliations:** Laboratório de Imunologia Clínica, Instituto Oswaldo Cruz, Fiocruz, Rio de Janeiro Brazil

**Keywords:** BCG vaccine, Tuberculosis, Monocyte, Apoptosis, Cytokine, Inflammation, Conditioned medium

## Abstract

**Background:**

Tuberculosis (TB) is the second greatest killer worldwide that is caused by a single infectious agent. For its control, studies of TB vaccines are needed. Since Bacillus Calmette-Guerin (BCG) is the only vaccine against TB currently in use, studies addressing the protective role of BCG in the context of inducible inflammatory mediators are urgently required.

**Methods:**

In this study, groups of HIV-negative adult healthy donors (HD; *n* = 42) and neonates (UV; *n* = 18) have been voluntarily enrolled, and BCG Moreau strain was used for the in vitro mononuclear cell infections for an initial period of 48 h. Subsequently, harvested conditioned medium (CM) was added to autologous resting cells for an additional 24, 48, and 120 h, and Annexin V, in conjunction with a vital dye, was then used for apoptosis detection. CM was also assayed for nitric oxide (NO), prostaglandin E2 (PGE_2_), leukotriene B4 (LTB_4_), interferon (IFN)-β, and transforming growth factor (TGF)-β1 levels. The *p* values were set up for any differences between two groups of individuals using Student’s *t*-test and considered significant when ≤ 0.05.

**Results:**

At 120 h, CM induced the highest apoptosis levels in both group studied, but necrosis was high in UV group only (*p*-value < 0.05). NO was released equally during BCG infection in both groups, but higher levels were found in HD when compared with UV group (*p*-value < 0.05). Overall, BCG Moreau triggered high PGE_2_, LTB_4_ and IFN-β productions in macrophages from the UV group (*p*-value ≤ 0.05), whereas the prostanoid PGE_2_ and TGF-β1 had an opposite pattern in the HD group.

**Conclusions:**

This study uncovers critical roles for endogenous compounds in the instruction of host macrophage cell death patterns. Understanding the regulation of human immune responses is critical for vaccine development and the treatment of infectious diseases. These findings shed new light on the potential condition for a booster immunization in individuals already vaccinated with BCG for TB protection, and further studies are warranted.

**Electronic supplementary material:**

The online version of this article (doi:10.1186/s12950-015-0105-0) contains supplementary material, which is available to authorized users.

## Background

Despite the continuous evolution of better management, new drug development, and additional diagnostic tools available in public health, tuberculosis (TB) still remains as a serious global problem. There were a total of 8.6 million new cases and 1.3 million deaths in 2012; TB is a leading killer of people living with HIV [1]. It is currently estimated that one third of the world population is infected with *Mycobacterium tuberculosis* and that 5–10 % of those infected will develop the disease during their life-time [2].

The current TB vaccine is *M. bovis* bacille Calmette-Guérin (BCG), and it has been employed for nearly a century to prevent the disease. However, while BCG is the most common and broadly used vaccine worldwide, its efficacy remains quite controversial: In endemic sites, the vaccine has evoked unpredictable outcomes [3]. Epidemiological studies point to BCG as affording more complete protection against the meningeal and miliary clinical forms of TB in children than against the pulmonary clinical form in adults (Reviewed by [4]). It is generally perceived that the BCG vaccine is not fully effective because of the absence of antigens commonly shared with *M. tuberculosis,* but also that the rapid removal of BGC via systemic immunity, primarily targeting atypical environmental mycobacteria, reduces the vaccine’s effectiveness over time. Therefore, more studies are required to better understand how BCG confers protection in humans.

Conventional studies have focused solely on the role of cytokines released by monocytes infected with BCG as a starting point for inducing immunity, such as the pro-inflammatory interleukin 1 beta (IL-1β) and tumor necrosis factor alpha (TNF-α) (Reviewed by [5, 6]). Thus, one of the issues raised concerns the additional soluble compounds that are potentially secreted by host cells during BCG infection. Also, there may be a later increase in those factors for the initiation of apoptosis, necrosis, necroptosis and pyroptosis. The action of inflammatory caspases, such as caspase-1, has shown promising results for the activation of IL-1β through an inflammasome mechanism during microbial pathogens interactions [7].

Our prior study supports the hypothesis that BCG induces distinct cell-death patterns during the maturation of the immune system [8]. In the study, we observed increasing apoptosis in BCG-stimulated monocytes from healthy, vaccinated adults, associated with a release of IL-1β and TNF-α, but not with metalloproteinase-9. Conversely, higher monocyte necrosis, but not apoptosis, was observed following the infection of umbilical vein cells from naïve neonates. This pattern was paralleled by different pro-inflammatory cytokine levels when compared to adults. In addition, necrosis has been defined as a mechanism used by virulent bacteria to exit macrophages, evade the host defenses, and disseminate; apoptosis has been associated with diminished pathogen viability [9, 10].

To address remaining critical issues beyond these premises, we have constructed a model of conditioned cell culture medium, i.e., culture supernatants obtained from host cells infected with BCG for 48 h, for the study of the factors secreted by those cells that potentially are responsible for monocyte cell-death during the context of infection. Our hypothesis has a basis in compelling evidence from one recent study where eicosanoid lipid intermediaries regulated the cell death program of human macrophages infected with *M. tuberculosis* [10]. Thus, a cross-sectional population study of samples from Brazil was the means to uncover critical aspects of the in vitro degree of apoptosis and necrosis induced by BCG Moreau in monocytes from individuals, whether sensitized (adults) or non-sensitized (neonates). Using this method, there is input for a better understanding of the protective factors afforded by BCG against TB that would help to identify the processes by which this protection is achieved, thus opening up a horizon for its future improved clinical applicability.

## Methods

### Study participants

Between November 2010 and August 2012, two groups of donors were enrolled for this study at the Gaffrée Guinle State University Hospital of Rio de Janeiro (HUGG): healthy donor adults (HD; a total of 42 individuals) from the blood bank (anonymous donation policy, but included individuals age ≥ 18-years old), and healthy mothers who participated in procedures to puncture umbilical cords and obtain blood samples from newborns’ umbilical vein (UV; a total of 18 neonates). Inclusion and exclusion criteria for those HIV-seronegative individuals are described elsewhere [8]. The HUGG Institutional Review Board approved this study under protocols #060/2009 and #089/2011. All participants in this study provided written informed consent.

### Mononuclear cells purification, culture, in vitro infection with BCG and cell death assay

The peripheral blood mononuclear cells (PBMC) and cord blood mononuclear cells (CBMC) were separated, cultured, and infected with *M. bovis* BCG Moreau strain for 48 h as previously described [8]. Cell cultures (1 × 10^6^) were used at a multiplicity of infection (MOI) of 2:1 (bacilli:mononuclear cell). The bacilli viability was promptly assessed by a commercially available LIVE/DEAD BacLight bacterial viability kit (Life Techonologies, Carlsbad, CA, USA) in combination with flow cytometry. Next, the cell-free supernatants, henceforth called conditioned medium (CM), were collected, passed through a 0.22 μm pore size filter (Millipore, Bedford, MA, USA), and instantly transferred (700 μl) to either autologous resting PBMC or CBMC (1 × 10^6^) for an additional 24, 48, and 120 h. This followed a protocol similar to that of Nagabhushanam and colleagues [11]. Subsequent to incubation, cells were labeled using an apoptosis detection kit as specified by the manufacturer (TACS, R&D systems, Minneapolis, MN, USA) and immediately analyzed in a flow cytometer device (FACScalibur, BD, San Jose, CA, USA). Those tubes designated as negative control (baseline) remained uninfected for the whole period of time. Other tubes designated as positive control consisted of cells subjected to a heating and boiling cycle just before staining in order to force cell necrosis [8]. A parallel process was used to establish the human acute monocytic leukemia cell line THP-1 (Kindly provided by Dr. M. C. V. Pessolani, FIOCRUZ, Brazil) in a monocyte-like state. Cells were grown, expanded, cultured in tubes (1 × 10^6^), and then infected with *M. bovis* BCG Moreau strain (MOI of 2:1) for 48 h. Next, cells were labeled and analyzed following the same apoptosis detection protocol as previously described.

### NO indirect detection by chemical test

A modified Griess diazotization reaction for microassay detection of the presence of organic nitrite compounds, such as nitric oxide (NO), was used in thawed cell-free supernatants (or frozen CM) as previously described [12].

### PGE_2_, LTB_4_, IFN-β, TGF-β1 detection by ELISA

Prostaglandin E2 (PGE_2_) and leukotriene B4 (LTB_4_), interferon (IFN)-β, and transforming growth factor (TGF)-β1 levels were determined in thawed cell-free supernatants (or frozen CM) using commercially available enzyme linked immunosorbent assay (ELISA) kits (Invitrogen Corp., Carlsbad, CA, USA; TFB Inc., Tokyo, Japan; and DuoSet, R&D systems, Minneapolis, MN, USA, respectively). The immunoassays were carried out according to the manufacturer instructions. The detection limits were 30 pg/ml (PGE_2_), 8 pg/ml (LTB_4_), 3.1 pg/ml (IFN-β), and 20 pg/ml (TGF-β1). For comparison purposes only, a thawed CM from BCG-infected mononuclears at 24 h was also included as an earlier time-point in both groups studied.

### Statistical evaluation

Data were analyzed using Instat Statistical software, version 3.06 for Windows (GraphPad Software Inc., La Jolla, CA, USA). The significance of the difference in levels of immune responses between the two groups of individuals was calculated using the Student’s *t*-test. The THP-1 cell-death levels were represented as the mean ± standard error of the mean (SEM). The *p*-value was scored and considered significant when ≤ 0.05.

## Results

### Cell-Death ratio

A better understanding of the pathways related to the in vitro cell-mediated immune responses in humans has been obtained for individuals, BCG-sensitized or not, which will assist in identifying the specific mechanisms that may confer protection against *M. tuberculosis*. To this end, and also based on our previous findings [8], we enrolled two groups of donors representing distinct immunity characteristics due to previous exposure to mycobacterial antigens. The first group consisted of healthy donor adults (HD) who had been already vaccinated with BCG Moreau during childhood. [Note: BCG vaccination in Brazil is universal after birth since 1967]. The second group consisted of naïve individuals (i.e. neonates) who had never been exposed to mycobacteria; Umbilical vein (UV) cells from the naive individuals were promptly collected after their delivery. After standard experimental procedures [8], CM resembling cell-free supernatant was generated from 48 h BCG Moreau-infected cells (bacilli viability > 90 %), and the autologous remaining resting cultures were further incubated with CM for additional 24, 48 and 120 h. Baseline cultures with no stimuli for a total of 168 h were used as a single negative control, since matched, earlier time-frames showed negligible differences. Cell-death events were analyzed to detect apoptosis and necrosis following an identical protocol described earlier [8]. Table 1 summarizes those findings.

**Table 1:** Specific cell-death (%) in monocytes of healthy donor (HD, *n* = 16) and umbilical vein (UV, *n* = 5) groups after transference of conditioned medium (CM)

	Apoptosis		Necrosis	
	HD	UV	HD	UV
Negative control	32.5 ± 4.2^a^	12.7 ± 2.1	12.2 ± 2.5	3.4 ± 1.1
CM 24 h	30.5 ± 4.5	14.8 ± 1.2	13.1 ± 2.0	2.4 ± 0.4
CM 48 h	36.1 ± 4.1	12.5 ± 1.8	12.7 ± 2.1	2.4 ± 0.5
CM 120 h	57.2 ± 3.6^b^	22.1 ± 1.4^b^	12.4 ± 1.7	9.0 ± 1.3^b^
Positive control^c^	31.8 ± 5.4	59.7 ± 4.7^b^	57.4 ± 5.3^b^	36.2 ± 4.8^b^

^a^Mean ± SEM;

^b^ *p* < 0.05, when compared to negative control (baseline);

^c^Heating samples was used to artificially induce necrosis

We observed a significant increase in apoptosis (*p*-value < 0.05) when CM was placed in contact with resting cells from both groups of individuals for an extended 120 h, as compared to the baseline, negative control. Moreover, only cells from UV group showed an increase in necrosis (*p*-value < 0.05) under the same conditions and incubation periods. Curiously, in shorter experimental time-frames, there were no cell-death compared with negative control in both groups studied (Table 1). In addition, and in opposition to the results described previously, the heating cycle caused an enhancement in apoptosis (*p*-value < 0.05) only in the UV group, although necrosis (*p*-value < 0.05) was the highest in both groups, as expected.

With the aim of clarifying whether most of the primary monocytes increase in cell-death levels occurred after the *M. bovis* BCG infection, we used a smaller set of experiments (*n* = 6) to test the THP-1 cell-line in our in vitro model. This step allowed us to confirm the previous results in primary mononuclear cell cultures (17.1 ± 1.0 and 24.7 ± 0.6; 6.5 ± 0.6 and 17.6 ± 0.8, at baseline and 48 h of BCG-infected THP-1 cell-line conditions for apoptosis and necrosis, respectively: *p*-values < 0.01). Thus, the THP-1 cell-line mirrored the cell-death patterns after BCG infection found particularly in the UV group studied.

Additionally, we attempted to better characterize the CM constituents in the next assays.

### Nitric Oxide levels

**Fig. 1:**
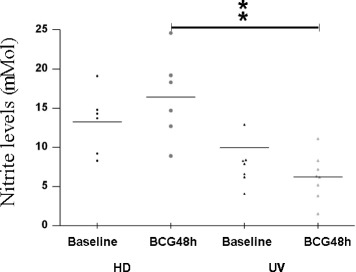
Nitric Oxide (NO) levels (mM) in healthy donor (HD; *n* = 6) and umbilical vein (UV; *n* = 8) groups representing baseline and 48 h of in vitro BCG Moreau infection in human mononuclears. Horizontal bars represent mean NO values in each condition. **p < 0.01

The production of NO by host innate immune cells is recognized to be a successful defense against infection, but the specific mechanism by which NO antagonize the pathogen, involving induction of apoptosis in macrophages that harbor the mycobacteria, is not completely understood [13, 14]. In addition, IL-1β, TNF-α, and mycobacterial cell wall components, along with T-cell-derived IFN-γ, may also induce NO release. Thus, NO released during 48 h-BCG infection in both PBMC and CBMC cultures (the frozen CM) were assayed, and the highest levels (*p*-value < 0.01) were found only in the HD when compared with UV group (Fig. 1).

### Inflammatory lipid mediators and cytokines levels

**Fig. 2:**
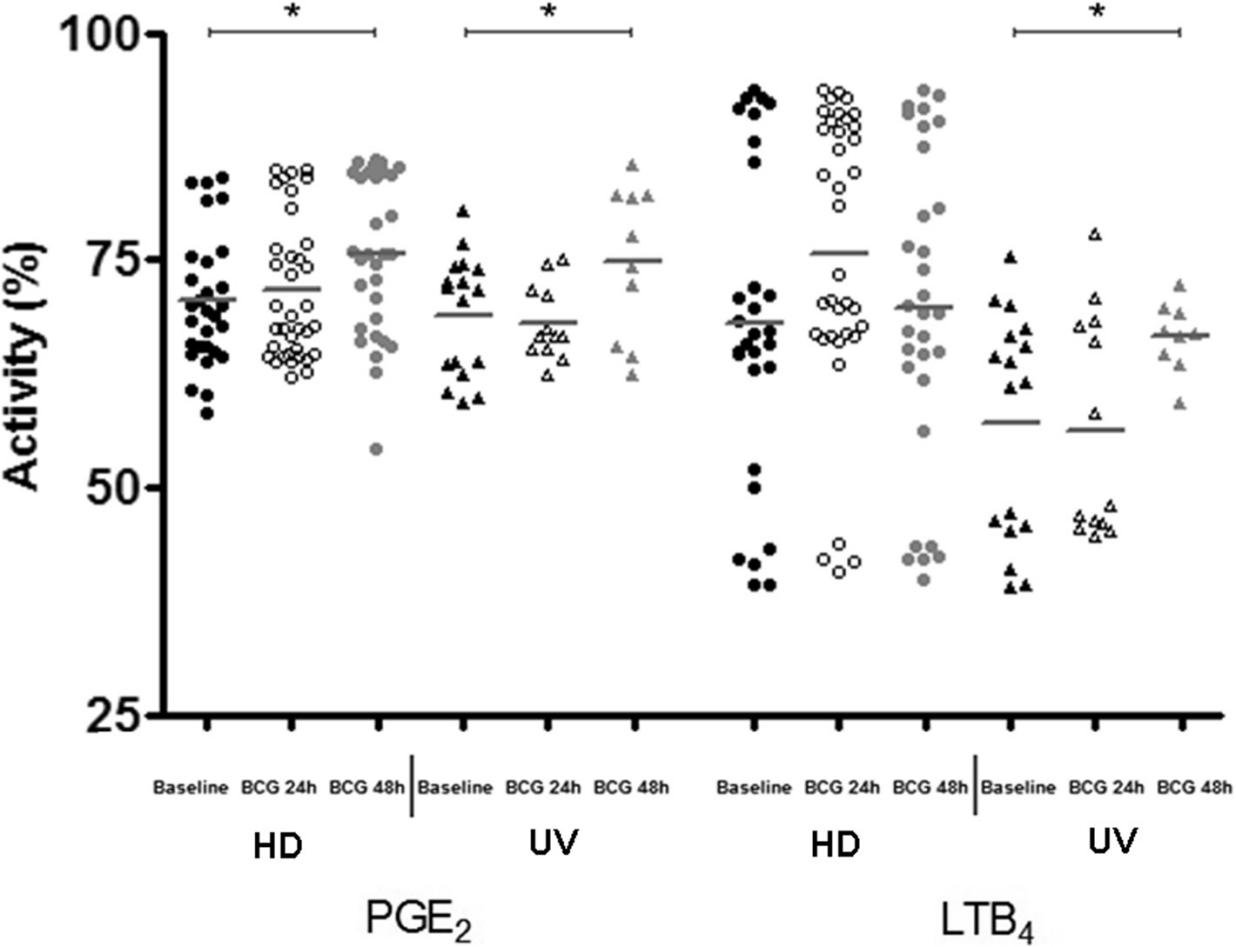
Prostaglandin E2 (PGE_2_) and leukotriene B4 (LTB_4_) activity levels (%) in healthy donor (HD; *n* = 36) and umbilical vein (UV; *n* = 17) groups representing baseline and different times of in vitro BCG Moreau infection in human mononuclears. Horizontal bars represent mean values in each condition. *p ≤ 0.05

**Fig. 3:**
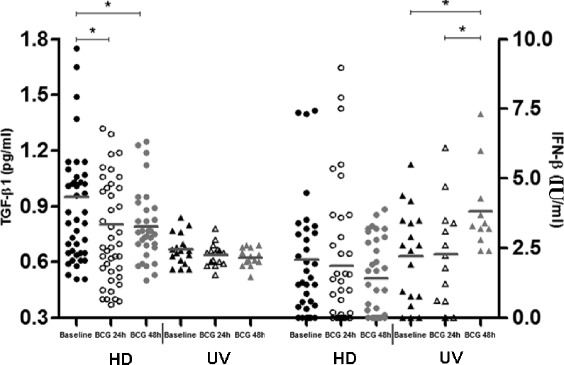
Transforming growth factor (TGF)-β1 levels (pg/ml) and Interferon (IFN)-β levels (IU/ml) in healthy donor (HD; *n* = 42) and umbilical vein (UV; *n* = 18) groups representing baseline and different times of in vitro BCG Moreau infection in human mononuclears. Horizontal bars represent mean values in each condition. *p ≤ 0.05

At an early encounter with noxious stimuli, the host produces a nonspecific inflammatory response, but a more specific immune response is believed to be modulated by two classes of molecules: eicosanoid lipid intermediaries, such as PGE_2_ and LTB_4_, and cytokines, synthesized by phagocytes [15]. A preceding study has associated increased prostanoid PGE_2_ levels with apoptosis, as well as high pronecrotic lipid mediators, such as LTB_4_ and necrosis [10]. Similarly, IFN-β levels were shown to be elevated in necrosis [16] and necroptosis as well [17]. Importantly, increased PGE_2_ concentrations have been found in TB patients compared to HD and/or latent TB infected patients [18]. In order to uncover critical aspects of cell-death modality induced by BCG Moreau and to corroborate any potential link of those ecosanoids and IFN-β with our in vitro cell-death system, competitive ELISA to the eicosanoids (Fig. 2), and ordinary ELISA to IFN-β and TGF-β1 were assayed (Fig. 3). When group means and dispersions for PGE_2_ in BCG-infected mononuclears were individually compared at 24 and 48 h to the baseline, a similar pattern was found in both groups studied (Fig. 2). However, the highest levels were achieved at 48 h only (*p*-value < 0.05). On the other hand, LTB_4_ was exclusively induced in UV group (*p*-value < 0.05; Fig. 2), whereas only a trend for higher levels after 24 h of the BCG-infected cells in the HD group was found (*p*-value = 0.08). In addition, the highest IFN-β levels (*p*-value ≤ 0.05) did follow the ecosanoids pattern for the UV group only, but TGF-β1 levels behaved as an opposite manner when compared to PGE_2_ in HD group, i.e. a drop in concentration after BCG-infected mononuclears (*p*-value ≤ 0.05; Fig. 3). Of note, when dispersions were compared between the 2 groups studied despite stimulus, UV group showed minimal range for TGF-β1 levels. Besides, in order to highlight overall variations and particular changes to those soluble mediators at the individual donor level, connecting lines were built and thus allowed the visualization for a trend to mostly individuals tested (Additional file 1: Figure S1).

## Discussion

Prophylactic medicine via vaccination is still the most cost effective means to reduce the spread of infectious diseases, and it also minimizes the burden on the health system. Because TB has imposed centuries-long threat to humans, the majority of the population in the tropics has been immunized with BCG in the infancy, but real protection against disease during adulthood has not yet been achieved. There is a long road ahead to better understand the entire clinical utility of BCG Moreau vaccine, and currently this task is our primary goal (Reviewed by [4]).

Here, consistent data have shown an enhancement of the in vitro apoptosis rate in monocytes from both adult and neonate individuals only after repeated long-term incubation with CM from autologous, infected BCG Moreau cultures that have been regularly used in a previous model. Hence, after a total of 48 h of infection, BCG stimulated a sufficient output of soluble factors in the CM, in both PBMC and CBMC cultures, to drive additional 120 h of autologous monocyte cell-death. This result, together with higher in vitro necrosis rate in the UV group, is generally in line with our previous data suggesting the distinct cell-death patterns induced by BCG [8]. That was associated both with higher NO released from the BCG-primed group, but not from the naïve individuals, and also by a distinct pattern of additional mediators and cytokines that found a close parallel to its respective cell-death outcome.

Reactive nitrogen intermediates, such as NO, have been reported to possess anti-mycobacterial activity, and it has long been established [13], as well as currently understood, that NO is crucial in the host response against intracellular pathogens [14]. Macrophages can directly inhibit the proliferation of mycobacteria by producing NO. In addition to the microbicidal properties, NO is also important as signaling molecules and therefore promotes macrophage activation and prominent apoptosis. This facilitates antigen presentation and helps to connect the innate to the adaptive immune responses [13, 14]. Hypothetically, the more apoptosis is induced by a vaccine against TB in host cells, the more effective is the response against *M. tuberculosis*. However, there is insufficient evidence to determine whether NO is involved in the defense against the TB bacillus in humans. Here, the higher NO levels released after BCG infection of mononuclears in the HD group, but not in the UV group, might denote indirectly a memory phenotype that remains active for years after priming, thus creating effective, long-lasting protection in those individuals. The balance of cytokines that efficiently upregulate NO production, such as IFN-γ, TNF-α and IL-1β, may play a critical role in the defense mechanism against TB [19].

As above stated, IFN-γ synergizes with TNF-α for NO production. Thus, our two previous in vitro studies corroborate with the present one through lower NO levels detected in neonate individuals: Virtually no IFN-γ production [20] or TNF-α levels [8] were found in those assessments. Therefore, studying human immune response using the BCG Moreau vaccine and also including the NO production may give us a better understanding of the TB pathogenesis as well.

Resistance or susceptibility to a given infection is a complex phenotype with a multifactorial trait, with several critical candidates playing an important role. As an example, arachidonic acid metabolites converted to the eicosanoids PGE_2_ and LTB_4_ are inflammatory mediators which are likely to be involved in apoptosis and necrosis episodes, respectively. Originally, PGE_2_ was described as the major prostanoid in the lung with an important antifibrotic role. On one hand, PGE_2_ mediates vasodilatation, increases vascular permeability, enhances pain perception by bradykinin and histamine, alters connective tissue metabolism, and enhances osteoclastic bone resorption. On the other hand, LTB_4_ causes the accumulation of inflammatory cells in the inflamed sites and degranulation of polymorphonuclear leukocytes [21]. The preferential synthesis of bioactive eicosanoids, mainly prostanoids, is a critical branch point for the innate antimycobacterial response of the infected macrophages. Distinct virulence determinants can manipulate macrophage inflammation toward both extremes, both inducing anti-inflammatory mediator production and also promoting pro-inflammatory TNF [10]. More importantly, PGE_2_ was recently defined as a pro-apoptotic host lipid mediator which protects against necrosis [9, 10]. In contrast, suppression of PGE_2_ synthesis by a pronecrotic mediator results in mitochondrial damage and inhibition of plasma membrane repair mechanisms, ultimately leading to the induction of necrosis. Thus, the balance between PGE_2_ and LTB_4_, presumably another pronecrotic lipid intermediary, may actually determine whether BCG-infected macrophages undergo apoptosis or necrosis, and this balance determines the outcome of infection. Accordingly, comparable to previous studies, we observed a significant pattern showing that PGE_2_ and LTB_4_ were highly correlated to the corresponding cell-death patterns; i.e. BCG-infected mononuclears induced PGE_2_ and apoptosis in both groups but induced LTB_4_ and necrosis in the UV group only. Finally, the absence of IL-1 decreases PGE_2_ in a model of *M. tuberculosis*-infected deficient mice, and the addition-of IL-1 or PGE_2_ reduces the bacteremia, whereas the TNF-induced LTB_4_ rises in wild-type animals [18]. Additionally, blocking of type I IFN signaling resulted in increased IL-1 and PGE_2_. Importantly, protection directly correlated with higher PGE_2_ and lower type I IFN and IL1Ra production. Likewise, PGE_2_ also inhibited type I IFN production in *M. tuberculosis*-infected human macrophages. These findings reveal that suppression of type I IFNs and their pro-bacterial activity is a main mechanism of IL-1- and PGE_2_-mediated host resistance against *M. tuberculosis*.

Similar to LTB_4_, IFN-β seems to be implicated earlier in necrosis [16] and more recently in necroptosis [17]. In fact, one of the mechanisms underlying enhanced susceptibility to bacterial infections is the stimulation of IFN-β production, as stated above, and the latter study showed a *Salmonella enterica*-induced macrophage death via necroptosis in a type I IFN-dependent manner. That novel cell-death modality is usually triggered by CD95 and TNF family of death receptors, and this programmed cell necrosis is distinct from apoptosis [22]. In another setting, pDCs were shown to be a major cell population producing IFN-β through toll-like receptor (TLR) signaling [23]. Generally, pDCs produce most IFN-β within 48 h after pathogen infection and then differentiate into mature DCs with enhanced antigen-presenting capability, after which they become refractory upon secondary stimulation and lose their IFN-β production ability. Our current data of highest IFN-β levels after that analogous period of BCG-infected mononuclears in the UV group again shows this cytokine to be well correlated with the necroptosis pattern shown by Robinson and colleagues [17]. However, in our system the exact underlying mechanism (necrosis vs. necroptosis) has not yet been fully elucidated.

TGFβ1 might act as an anti-inflammatory arm prone to damper Th1 immune response. Actually, in line with the current data, our very recent study also failed to find any Th3 cell phenotype induced by the BCG vaccine in the sensitized adult cohort: Comparable TGFβ1 levels were found regardless of the stimulus employed [24]. That study originally established that the BCG Moreau vaccine induced a significantly elevated IFN-γ/IL-10 ratio only in the HD group, confirming a polarized Th1 pattern in vitro.

Based on those previous findings, our current primary results suggest that apoptosis was again induced in monocytes, probably via TNF and/or IL-1β in adults, but not in neonates [8]. Of note, IL-1β drives PGE_2_ synthesis in infected human macrophages [18]. However, we could not rule out a role for other candidates, such as ATP, TWEAK, TRAMP, TRAIL, and CD95, since there is a long list of potential candidates for apoptosis induction in our system, particularly for the new data regarding naïve individuals [25].

Taking together, one could conclude that the apoptosis pattern found in resting monocytes from both immunized and naïve groups correlates well with high pro-inflammatory cytokine levels induced in BCG-infected mononuclears. However, necrosis data supports previous findings in neonates only, but additional studies are warranted to rule out other mechanisms such as pyroptosis or necroptosis. These findings support the hypothesis that BCG Moreau strain induces a milieu of soluble factors, which further stimulate distinct cell-death patterns regarding the maturation of the immune system. This pattern may set the stage for a subsequent anti-mycobacterial immune response, which could have profound effects during vaccination.

A possible unifying hypothesis on the central role of cell-death modality in TB pathogenesis is that lesser apoptosis levels in BCG-infected monocytes of neonates could predispose those individuals to the development of inflammatory disease due to lack of protective and specific immune response. This is because only the phagocytosis of apoptotic cells leads to the resolution of inflammation [26]. Conversely, the progress to necrosis could predispose those individuals to the development of disease, since virulent *M. tuberculosis* inhibits apoptosis and triggers necrosis of host macrophages, which delays the initiation of adaptive immunity [9, 10]. Thus, BCG immunization is the only pathway for increasing the immune response against *M. tuberculosis*. On the other hand, BCG-vaccinees show a balance between regulatory and effective immune responses, and a breakdown in this equilibrium would be crucial for TB development.

Despite some tantalizing clues and frequent overstatements, the underlying immunological mechanisms by which the BCG Moreau vaccine evokes protective immune response against *M. tuberculosis* continues to be poorly understood. The central puzzle that remains to be deciphered is: How exactly does the BCG immunization confer partial protection in the vaccinees?

## Conclusion

In this study, BCG-infected macrophages from a naïve group triggered PGE_2_, LTB_4,_ and IFN-β levels, but BCG triggered copious amount of NO and PGE_2_ in adults only. Accordingly, BCG elicited a cell death modality leading to cytolysis in infected macrophages from the naïve group only. In striking contrast, apoptosis was found in the same system in both groups studied. Taken together, our data is in line with other studies demonstrating critical roles for endogenous compounds, such as NO, PGE_2_, LTB_4_, IFN-β and TGF-β1, in the regulation and induction of host macrophage cell death patterns and their relevant role in the modulation of inflammation. A better understanding of the factors that protect against TB will help us identify the processes by which this protection is achieved, thus opening up a horizon for its future clinical applicability. Ultimately, because prevention is still one of the best approaches to manage infectious diseases, this leads to the development of improved prophylactic vaccines for TB that, together with infectious diseases in general, disproportionately affects poor and marginalized populations.

## Additional file

Additional file 1: Figure S1. Connecting lines for (A) Prostaglandin E2 (PGE_2_) and (B) leukotriene B4 (LTB_4_), in activity levels (%), and (C) Transforming growth factor (TGF)-β1 levels, in pg/ml, and (D) Interferon (IFN)-β levels, in IU/ml, in healthy donor (HD; *n* = 20) and umbilical vein (UV; *n* = 6) groups representing baseline and different times of in vitro BCG Moreau infection in human mononuclears. (PPT 51 kb)

## Abbreviations

TB: Tuberculosis
BCG: Bacillus Calmette-Guerin
HD: Healthy donors
UV: Umbilical vein
HIV: Human immunodeficiency virus
CM: Conditioned medium
PBS: Phosphate buffered saline
DMSO: Dimethyl sulfoxide
ELISA: Enzyme linked immunosorbent assay
PGE_2_: Prostaglandin E2
LTB_4_: Leukotriene B4
IFN: Interferon
TGF: Transforming growth factor
NO: Nitric oxide
IL-1: Interleukin-1
IL-10: Interleukin-10
TNF: Tumor necrosis factor
pDC: Plasmacytoid dendritic cell
TLR: Toll-like receptor
PBMC: Peripheral blood mononuclear cell
CBMC: Cord blood mononuclear cell
FACS: Fluorescence-activated cell sorting
ATP: Adenosine triphosphate
TWEAK: TNF-related weak inducer of apoptosis
TRAMP: TNF receptor-related apoptosis-mediating protein
TRAIL: TNF-related apoptosis-inducing ligand
SEM: Standard error of the mean
