# Supplementary figures and images for: The role of host soluble inflammatory mediators induced by the BCG vaccine for the initiation of in vitro monocyte apoptosis in healthy Brazilian volunteers
Source: J Inflamm (Lond). 2015 Oct 29;12:60. doi: 10.1186/s12950-015-0105-0 (PMC4625933; doi:10.1186/s12950-015-0105-0)

## Slide 1
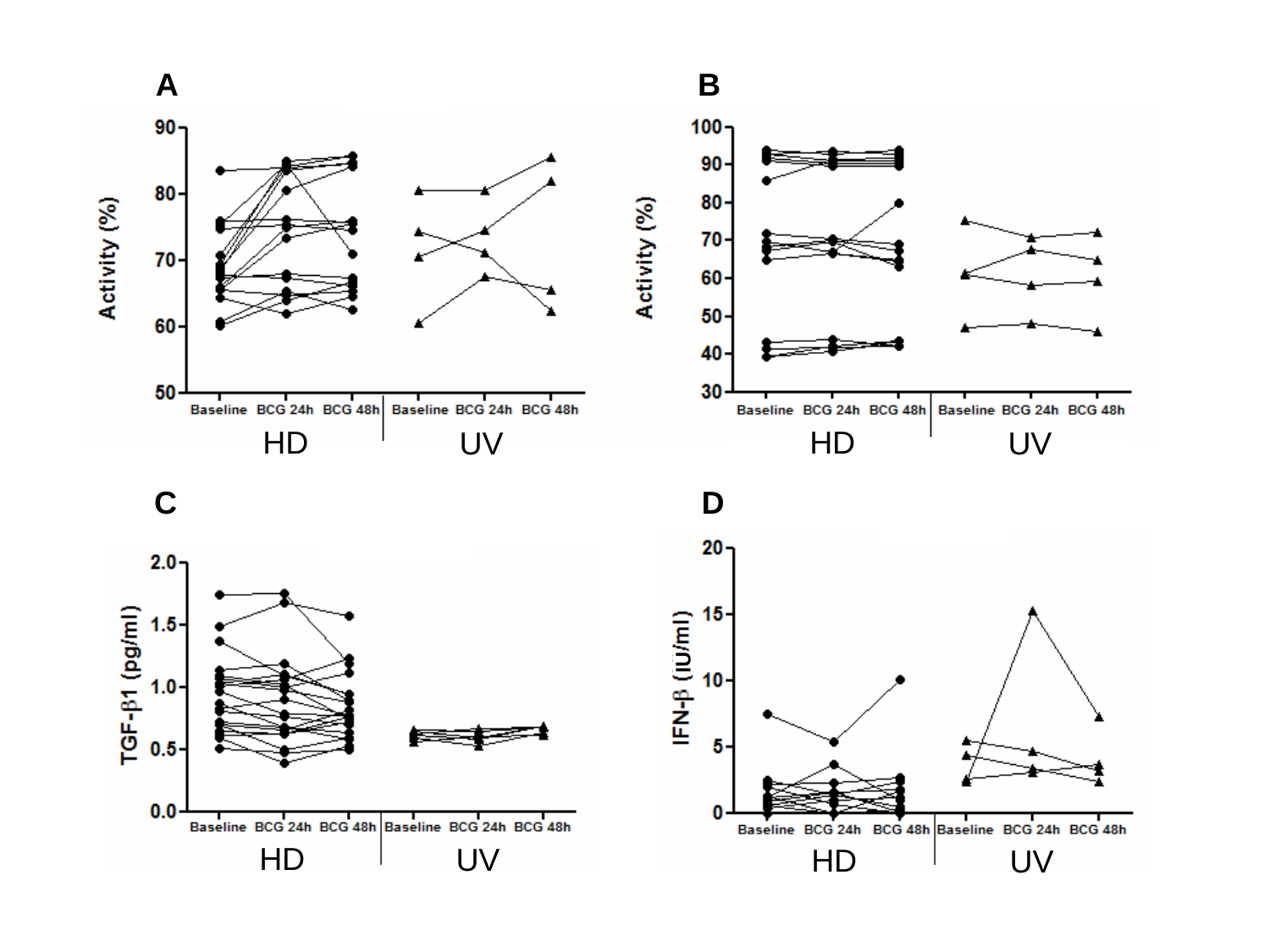

B
HD
UV
A
HD
UV
D
HD
UV
C
HD
UV
IU

Supplement: Additional file 1: Figure S1. — Connecting lines for (A) Prostaglandin E2 (PGE2) and (B) leukotriene B4 (LTB4), in activity levels (%), and (C) Transforming growth factor (TGF)-β1 levels, in pg/ml, and (D) Interferon (IFN)-β levels, in IU/ml, in healthy donor (HD; n = 20) and umbilical vein (UV; n = 6) groups representing baseline and different times of in vitro BCG Moreau infection in human mononuclears. (PPT 51 kb) [file 12950_2015_105_MOESM1_ESM.ppt]
